# APUM23, a PUF family protein, functions in leaf development and organ polarity in *Arabidopsis*

**DOI:** 10.1093/jxb/ert478

**Published:** 2014-01-21

**Authors:** Tengbo Huang, Randall A. Kerstetter, Vivian F. Irish

**Affiliations:** ^1^Department of Plant Biology and Pathology, Waksman Institute, Rutgers, State University of New Jersey, 190 Frelinghuysen Road, Piscataway, NJ 08854, USA.; ^2^Department of Molecular, Cellular and Developmental Biology, Yale University, New Haven, CT 06520-8104, USA.; ^3^Department of Ecology and Evolutionary Biology, Yale University, New Haven, CT 06520-8106, USA.

**Keywords:** APUM23, *Arabidopsis*, *KANADI*, leaf development, polarity, PUF.

## Abstract

The normal biological function of leaves, such as intercepting light and exchanging gasses, relies on proper differentiation of adaxial and abaxial polarity. *KANADI* (*KAN*) genes, members of the GARP family, are key regulators of abaxial identity in leaf morphogenesis. This study identified a mutant allele (*apum23-3*) of *APUM23*, which encodes a Pumilio/PUF domain protein and acts as an enhancer of the *kan* mutant. *Arabidopsis* APUM23 has been shown to function in pre-rRNA processing and play pleiotropic roles in plant development. The *apum23-3* mutant also synergistically interacts with other leaf polarity mutants, affects proliferation of division-competent cells, and alters the expression of important leaf polarity genes. These phenotypes show that APUM23 has critical functions in plant development, particularly in polarity formation. The *PUF* gene family is conserved across kingdoms yet it has not been well characterized in plants. These results illuminating the functions of *APUM23* suggest a novel role for *PUF* genes in *Arabidopsis* leaf development.

## Summary

This study characterized novel functions of APUM23, a PUF family protein, in *Arabidopsis* development. APUM23 functions in rRNA biosynthesis and has pleiotropic roles, influencing cell division and adaxial–abaxial polarity.

## Introduction

The leaves of many plant species exhibit differences between their adaxial (dorsal) and abaxial (ventral) surfaces (Telfer and Poethig, 1994). Proper specification of adaxial and abaxial identity is required for the formation and function of leaves. In *Arabidopsis thaliana*, adaxial identity is specified by the class III homeodomain-leucine zipper (HD-ZIPIII) genes (McConnell *et al.*, 2001; Emery *et al.*, 2003; Prigge *et al.*, 2005), Myb and LOB domain transcription factors *ASYMMETRIC LEAVES1* (*AS1*) and *AS2* (Lin *et al.*, 2003; Xu *et al.*, 2003), and *trans*-acting short-interfering RNA (ta-siRNA) (Allen *et al.*, 2005; Williams *et al.*, 2005; Hunter *et al.*, 2006), whereas on the abaxial side, *KANADI* genes (Eshed *et al.*, 2001, 2004; Kerstetter *et al.*, 2001), *AUXIN RESPONSE FACTORS* (*ARF*) *ETTIN* (*ETT/ARF3*) and *ARF4* (Pekker *et al.*, 2005), *YABBY* genes (Sawa *et al.*, 1999; Siegfried *et al.*, 1999; Eshed *et al.*, 2004), *LITTLE ZIPPER* (*ZPR*) genes (Wenkel *et al.*, 2007), and microRNA165/166 (Bao *et al.*, 2004; Kidner and Martienssen, 2004; Mallory *et al.*, 2004) play important roles.

The *HD-ZIPIII* gene family consists of members such as *REVOLUTA* (*REV*), *PHABULOSA* (*PHB*), and *PHAVOLUTA* (*PHV*). These genes encode proteins with partially redundant functions. Gain-of-function of one of these genes or simultaneous downregulation of all three genes results in radial cotyledons and leaves (McConnell and Barton, 1998; Emery *et al.*, 2003). Similarly to the *HD-ZIPIII* gene family, *KANADI* genes also have overlapping functions. Mutations in any single *KAN* gene have relatively mild defects in leaf polarity (Eshed *et al.*, 2001; Kerstetter *et al.*, 2001; McAbee *et al.*, 2006). However, if several of these genes are non-functional, the resulting plants exhibit strong defects associated with the loss of abaxial identity. For instance, the *kan1 kan2* double mutant has reduced leaf blade expansion and develops ectopic outgrowths on the abaxial side of the leaf. More dramatically, in the *kan1 kan2 kan3* triple mutant, leaves are almost fully radialized and adaxialized (Eshed *et al.*, 2004).

*HD-ZIPIII* and *KANADI* are thought to act antagonistically (Kidner and Timmermans, 2007). The *HD-ZIPIII* gene *PHB* is expressed abaxially in *kan1 kan2 kan3* triple mutants and its adaxial expression is reduced when *KAN2* is expressed throughout the leaf primordia (Eshed *et al.*, 2004). In addition, *KAN1* also represses *AS2* by directly interacting with its promoter and regulating its transcription (Wu *et al.*, 2008). These results demonstrate that the interactions of key adaxial–abaxial genes are critical for leaf polarity establishment and subsequent blade expansion.

This study identified a new regulator of leaf polarity, *APUM23*, which interacts with major polarity genes including *KANADI*, *AS2*, and *REV*. *APUM23* encodes a protein belonging to the PUF RNA-binding protein family (Abbasi *et al.*, 2010). *Drosophila* Pumilio is a founding member of this family and is required for the establishment of anterior–posterior polarity (Murata and Wharton, 1995) and stem cell maintenance (Lin and Spradling, 1997; Forbes and Lehmann, 1998) through translation inhibition. PUF proteins specifically bind to nanos response element sequences in the 3′-untranscribed region of target mRNAs (Zamore *et al.*, 1999; Wang *et al.*, 2001; White *et al.*, 2001) and usually function in a complex with other RNA-binding proteins such as Nanos (Sonoda and Wharton, 1999) and Brat (Sonoda and Wharton, 2001). In *Arabidopsis*, more than 20 putative *PUF* genes (*APUMs*) have been predicted by various studies (Francischini and Quaggio, 2009; Abbasi *et al.*, 2010; Tam *et al.*, 2010). Biochemical experiments have shown that several *Arabidopsis* PUF proteins are able to bind to *Drosophila* nanos response element sequences and the 3′-untranscribed region of mRNAs from *Arabidopsis* genes involved in shoot stem cell maintenance, such as *WUSCHEL* and *CLAVATA1* (Francischini and Quaggio, 2009), suggesting a evolutionarily conserved mechanism of PUF protein action. However, in contrast to the products of other *APUM* genes, APUM23 was found to be localized to the nucleolus and involved in pre-rRNA processing and rRNA maturation (Abbasi *et al.*, 2010). This distinct function of APUM23 is probably due to its unique structure in the PUF RNA-binding domains (Tam *et al.*, 2010).

APUM23 has been shown to play important roles in various aspects of plant growth (Abbasi *et al.*, 2010). The current work shows that APUM23 has a previously undescribed role in regulating the activity of division-competent cells and the establishment of organ polarity. The results suggest that APUM23 is important for organ growth and pattern formation in *Arabidopsis*.

## Materials and methods

### Plant materials

The *apum23-3*, *kan1-11*, and *kan2-5* plants used in this study were in the Columbia (Col) background. *as1-1* (CS3774, in Col background), *as2-2* (CS3118, in ER background), and *rev-1* (CS3826, in No background) were obtained from the *Arabidopsis* Biology Resource Center (ABRC). The *kan1-11* and *kan2-5* alleles have been described previously (Wu *et al.*, 2008). All plants were grown at 22 °C under long-day conditions (16/8 light/dark). Double mutants *apum23-3 as1-1*, *apum23-3 as2-2*, and *apum23-3 rev-1* were generated by initially crossing two parental lines followed by backcrossing the F1 with both parents. The progeny of the backcross was selected for parental phenotypes. The selected plants were self-pollinated and the double mutants were identified from the next generation. F2 progeny from the self-pollinated F1 plants was also examined. The double mutants were found at the ratio of 1/16 or less, and no enhancement of double mutant phenotypes (or other novel phenotypes) was identified in the F2 plants, ruling out background effects on the phenotypes of the double mutants. In order to make the *apum23-3 kan1-11 kan2-5* triple mutant, *apum23-3* was first pollinated with *kan1-11 kan2-5*. The F1 then was backcrossed to *kan1-11 kan2-5*. In the progeny of the backcross, plants homozygous for *kan1-11* and *apum23-3* and heterozygous for *kan2-5* were maintained, and the triple mutants were selected in their progeny by genotyping. The names of the double and triple mutants are abbreviated as *kan1 kan2*, *apum23 as1*, *apum23 as2*, *apum23 rev*, and *apum23 kan1 kan2*.

### Anatomical analysis

Petioles of 21-d-old plants were fixed in 2% glutaraldehyde in 25mM sodium phosphate buffer (pH 6.8) with 0.1% Triton X-100 overnight at 4°C. Samples were washed in the same sodium phosphate buffer, dehydrated through an ethanol series, and embedded in wax blocks. Sections (8 μm) were made on a Jung Biocut microtome and stained with 0.1% toluidine blue.

### Molecular identification of the enhancer of *kan1 kan2*

The enhancer of *kan1 kan2* mutant was identified by a map-based approach. The single mutants were first crossed with L *er* plants. The F2 generation segregating the enhancer phenotype was used as the mapping population, in which 929 mutant plants were identified. Genomic DNA was isolated from these mutants and utilized for mapping by using InDel and cleaved-amplified polymorphic sequence (CAPS) markers based on the Cereon L *er/*Col SNP database (TAIR; http://www.arabidopsis.org). These molecular markers localized the mutation at the bottom of chromosome I, in an approximate 17-kb region containing 13 genes. Sequencing six candidate genes revealed a G→A change that disrupts the intron splicing in At1g72320 (*APUM23*). In order to confirm the molecular identity of the enhancer as an allele of *APUM23*, a 6.7-kb genomic DNA fragment containing the entire At1g72320 gene was released from the BAC T10D10 by restriction digestion with *Sal*I and *Acc*65I (Fermentas, Hanover, MD, USA) and cloned in the binary vector pCAMBIA1300 and transformed into the enhancer mutant using the floral dip method (Clough and Bent, 1998). A control transformation was also performed with the empty pCAMBIA1300 vector. The T1 transgenic plants were selected on 1/2 MS medium containing 30mg l^–1^ hygromycin. In addition, a T-DNA line (SAIL_757_B08; *apum23-1*; Abbasi *et al.*, 2010) was also obtained from the ABRC. The T-DNA insertion in this line was confirmed by sequencing.

### Genetic complementation in yeast

Yeast Magic Marker *nop9* strain (BY4743) was obtained as a heterozygous diploid knock out (open biosystems, Huntsville, AL, USA). *APUM23* full-length cDNA was amplified from the BAC clone U09300 (obtained from ABRC) with primers GATEARW1N.f (5′-ATGGTTTCTGTTGGTTCTAAATCATTG-3′) and GATEA RW1N.r (5′-ACGTCTCAAATTCTCATTTTATTTGAATGCCG-3′). The PCR product was cloned into the pCR8 vector (Invitrogen, Eugene, OR, USA) and subcloned into the yeast expression vector BG1805 (a gift from Beth Grayhack) containing the GAL1 promoter and URA3 marker by GATEWAY cloning strategy (Invitrogen). The resulting plasmid (BG1805*-APUM23*), as well as the wild-type *NOP9* in the same vector (BG1805*-NOP9*), which was purchased (open biosystems), was transformed into the *nop9*/+ diploid strain. An empty BG1805 vector was also transformed separately as a control. The transformed cells were sporulated and spread on Magic Medium (Pan *et al.*, 2004) to select haploid cells that survived in the absence of uracil. The transformants were further confirmed by several sets of primers: NOP9A (5′-GTTTTCAAACTTGCTAGGCTGTATC-3′) and NOP9B (5′-CCAGTTCTTCTCTATCTAGCACACC-3′) were used to confirm the knock out of *NOP9* gene in the *nop9* genome, whereas NOP9A and kanB (5′-CTGCAGCGAGGAGCCGTAAT-3′) were used to detect the *kanMX* insertion that knocks out *NOP9*. Primers BNOP9.f (5′-CAAACCTTCAAATGAACGAATCAA-3′) and BNOP9.r (5′-agactcttcttgctggatgtgctc-3′) confirmed the presence of BG1805*-NOP9* in the haploid cells and BARW1.f (5′-GCGAAGCGATGATTTTTGATCTAT-3′) and BARW1.r (5′-CTTCCTACGCATTCCCTTATTCCT-3′) detected BG1805*-APUM23* in the haploid cells.

Haploid cells containing either *NOP9* or *APUM23* were inoculated in the liquid Magic Medium and grown in 30 °C until OD600 reached 0.6. The culture was serially diluted in sterile water and spotted on solid Magic Medium. The plates were incubated at 30 °C for 2 d for analysis.

### Examination of β-glucuronidase (GUS) reporter activities

*CYCB1:db:GUS* (Harrar *et al.*, 2003), p*KAN1:GUS*, and p*AS2:GUS* (Wu *et al.*, 2008) were introduced in *apum23-3* via crossing. GUS staining was performed according to Senecoff *et al.* (1996) with modifications. Plants were fixed in 80% acetone at –20 °C for 20min, then stained with 2mM X-Gluc in GUS-staining buffer (9mM potassium ferrocyanide and potassium ferricyanide) for 1h at 37 °C. After removing the chlorophyll with an ethanol series, the young leaves or roots were dissected, mounted in 10% glycerol, and observed under microscope.

### Reverse-transcription PCR

Total RNA (2 μg) extracted from 10-d-old seedlings using TRIzol Reagent (Invitrogen) was treated with DNase I (Fermentas) and reverse transcribed with Superscript III (Invitrogen), and 1 μl of a 10-fold dilution was used as template for PCR. To quantify the unprocessed pre-rRNA, the cleavage site of 35S pre-RNA was specifically amplified by primers U1 (5′-CGTAACGAAGATGTTCTTGGC-3′) and U2 (5′-ATGCGTCCCTTCCATAAGTC-3′). The primer pair UBC.f (5′-TCAAGAGGTTGCAGCAAGA-3′) and UBC.r (5′-CTTTGCTCAACAACATCACG-3′) was employed to amplify a ubiquitin conjugating enzyme gene as a control. The PCR conditions were 94 °C for 15 s, 52 °C for 15 s, and 72 °C for 30 s, for 33 cycles. The DNA band intensity was measured by using a KODAK Molecular Imaging Software 4.0 (Eastman KODAK Company, Rochester, NY, USA). Bands were normalized using Gaussian curve with background subtraction. Mean intensities and standard error of the mean were calculated from three independent biological samples.

### Quantitative real-time PCR

Young leaf primordia of Col and *apum23-3* were dissected from seedlings that were grown on half-strength MS plates for 2 d after the emergence of the first two leaves. Total RNA was extracted from the primordia and reverse transcribed as already described. Quantitative real-time PCR (qRT-PCR) utilized Power SYBR Green PCR Master Mix (Applied Biosystems) for amplification. Primers used in qRT-PCR are listed in Supplementary Table S1 available at *JXB* online. Changes in gene expression were calculated from three biological replicates using the 2^–ΔΔCt^ method (Livak and Schmittgen, 2001). The relative mRNA levels were normalized to the expression of *GAPC2* (Husar *et al.*, 2011; Mafra *et al.*, 2012).

## Results

### Identification of an enhancer of the *kan1 kan2* leaf polarity phenotype

**Fig. 1.**
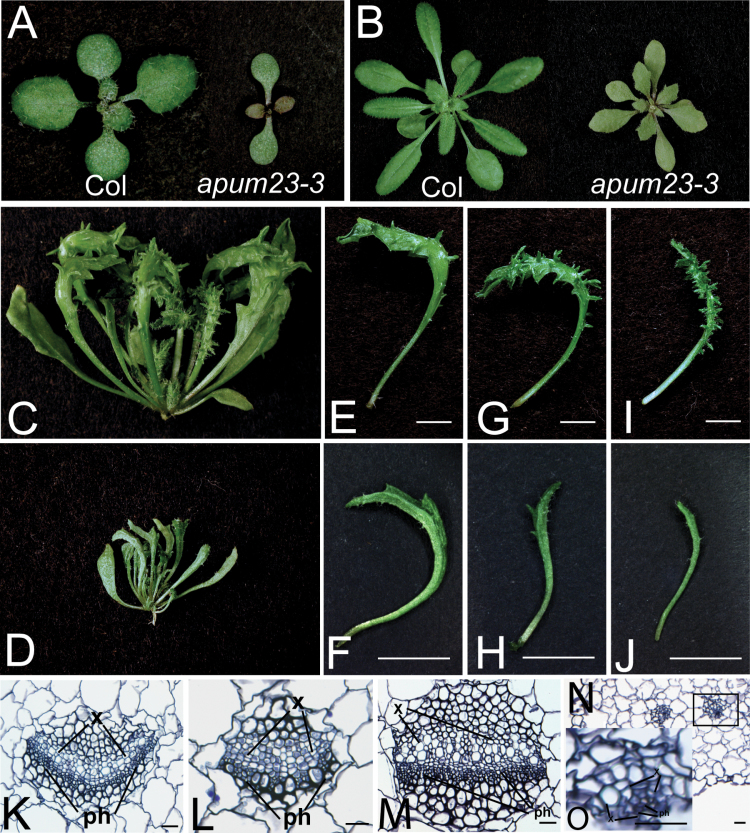
*apum23-3* enhances the *kan1 kan2* double mutant phenotype. (A–D) 10-d-old wild type (Col and apum23-3) (A), 21-d-old Col and *apum23-3* (B), 30-d-old *kan1 kan2* (C), and *apum23 kan1 kan2* (D). (E–J) Individual leaves 5, 7, and 9 of *kan1 kan2* (E, G, I) compared with those of *apum23 kan1 kan2* (F, H, J): note that blade expansion on *kan1 kan2* is reduced in the triple mutant. (K–O) Sections of petioles to examine the vascular structures of Col (K), *apum23-3* (L), *kan1 kan2* (M), and *apum23 kan1 kan2* (N, O) plants: O is a higher magnification of the vascular tissue in the black box in N, which shows the dramatic adaxialization in the triple mutant indicated by the xylem-surrounding-phloem phenotype. ph, phloem; x, xylem. Bars, 5mm (E–J) and 20 μm (K–O) (this figure is available in colour at *JXB* online).

An EMS mutagenesis screen was carried out to identify enhancers of the *kan1-11 kan2-5* (abbreviated as *kan1 kan2*) double mutant*. kan1 kan2* plants have upward-curling leaves with ectopic outgrowths on the abaxial side (Eshed *et al.*, 2001; Fig. 1C–I). One enhancer mutation was identified that enhanced *kan1 kan2* by having rosette leaves with reduced blade expansion (Fig. 1D–J). This defect became more severe as leaf number increased. For instance, leaf 9 of the triple mutant was almost completely radialized indicating a dramatic loss of leaf polarity (Fig. 1J). In addition, the abaxial outgrowths of the *kan1 kan2* mutant were also reduced in the triple mutant.

In order to further characterize the mutant phenotypes, the internal structure of the leaf petioles was analyzed. Distinct from the relatively normal midveins of *kan1 kan2* double mutant (Fig. 1M), transverse sections showed that the triple mutant had a disorganized vascular pattern consisting of very few phloem cells. These phloem cells were surrounded by adjacent xylem tissues suggesting a strong loss of abaxial identity (Fig. 1N, O). This enhancer is a recessive mutation and the single mutant had elliptical, flat and serrated leaves with a greyish green adaxial side (Fig. 1A, B; see also Fig. 6A). In contrast to the phenotype seen in the triple mutant, the enhancer mutant had very subtle vascular defects with a midvein structure similar to that observed in the wild type Col (Fig. 1K, L).

### The *kan1 kan2* enhancer is a mutant allele of *APUM23*

**Fig. 2.**
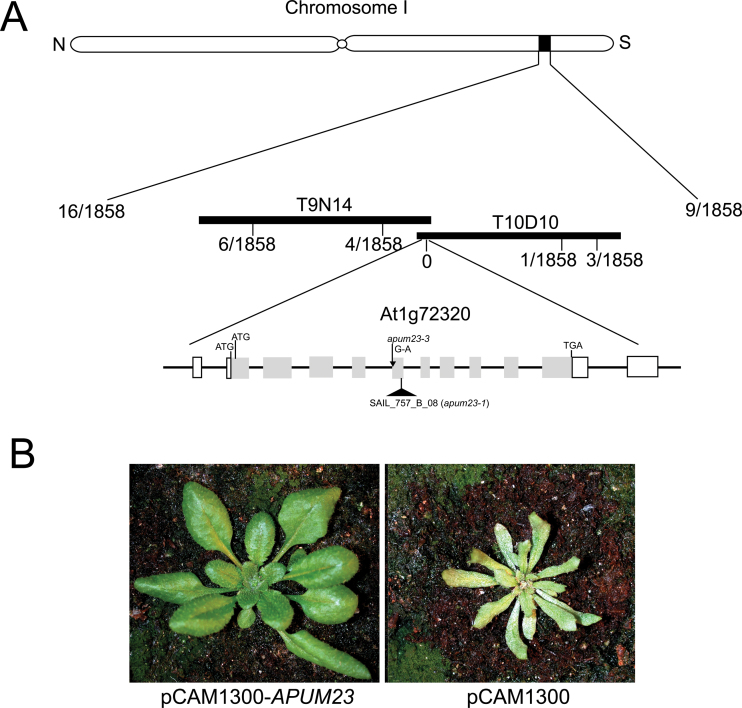
Molecular identification of *apum23-3*. (A) Positional cloning and gene structure of *APUM23*: solid black lines represent two bacterial artificial chromosomes (T9N14 and T10D10) containing *APUM23*. The recombination frequency at each molecular marker is shown as number of recombinations/number of chromosomes analysed. The genomic structure of *APUM23* (At1g72320) is illustrated with grey solid bars representing exons and black lines representing introns. *APUM23* has two non-coding exons at the 5′- and 3′-ends, which are represented by hollow bars. The lesion in *apum23-3* and the SAIL T-DNA line (*apum23-1*) are also shown. (B) Transgenic plant containing the *APUM23* genomic sequence transformed into the *apum23-3* mutant background shows complete rescue of the *apum23* mutant phenotype (left), whereas transgenic plants containing the empty vector pCAMBIA1300 still show the mutant phenotype (right) (this figure is available in colour at *JXB* online).

The enhancer mutation was mapped to a region containing 13 genes at the bottom of chromosome I, corresponding to the overlap of bacterial artificial chromosomes T10D10 and T9N14. Sequencing of six genes in this region in the enhancer mutant background revealed a single nucleotide mutation (G→A) in *APUM23* (At1g72320; Abbasi *et al.*, 2010) that disrupted the 3′-splice site of intron 5 in this gene (Fig. 2A). This suggested that this lesion may account for the enhancer phenotype. The molecular identity of the enhancer was verified by transgene complementation. A 6.7-kb genomic DNA fragment including the wild-type *APUM23* was transformed into the enhancer mutant line and resulted in transgenic plants with a wild-type phenotype, while plants carrying the empty vector did not show complementation (Fig. 2B). Moreover, T-DNA insertion alleles of *APUM23* also showed similar phenotypes to those of the enhancer (Abbasi *et al.*, 2010; Supplementary Fig. S1 available at *JXB* online), which further confirmed that the *kan1 kan2* enhancer mutation corresponds to a new allele of *APUM23*, named *apum23-3*.

### *apum23-3* interacts synergistically with mutants of genes specifying adxaxial identity

**Fig. 3.**
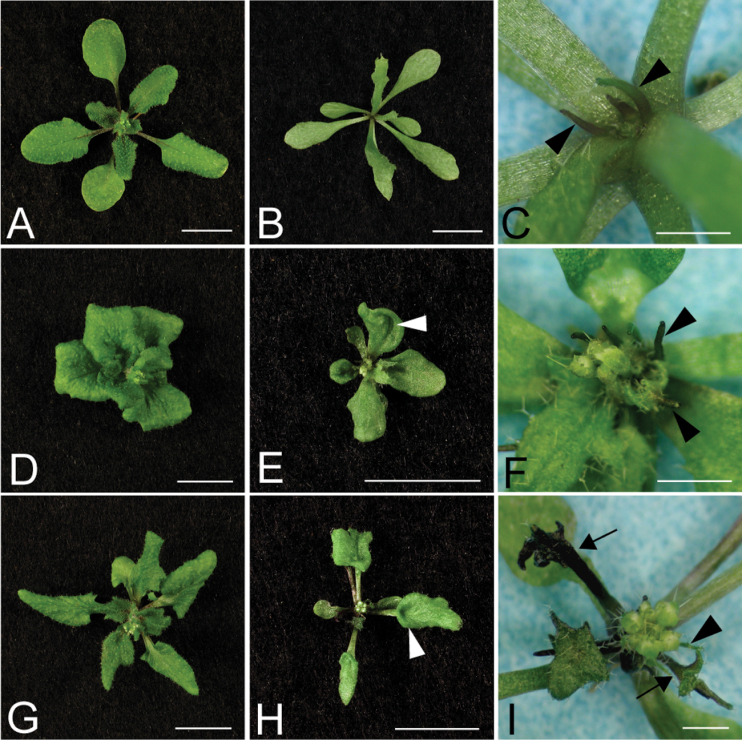
*apum23-3* interacts with adaxial polarity mutants. Phenotypes of 21-d-old *rev-1* (A), *as1-1* (D), *as2-2* (G), *apum23 rev* (B), *apum23 as1* (E), and *apum23 as2* (H). C, F, and I are higher magnifications of B, E, and H, respectively. White arrowheads in E and H indicate the trumpet-shaped leaves in *apum23 as1* and *apum23 as2*; black arrowheads in C, F, and I show the pin-shaped structures in the double mutants; black arrows in I show the branched radialized structures in *apum23 as2*. Bars, 1mm (C, F, I) and 1cm in all other panels (this figure is available in colour at *JXB* online).

**Fig. 4.**
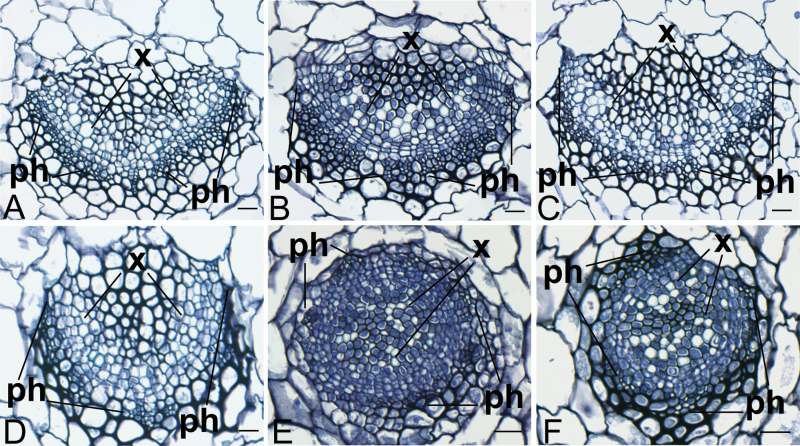
Vascular phenotypes of leaf petioles of adaxial polarity mutants and double mutants with *apum23-3*. Transverse sections of leaf petioles showing vascular organization in 21-d-old *rev-1* (A), *as1-1* (B), *as2-2* (C), *apum23 rev* (D), *apum23 as1* (E), and *apum23 as2* (F) plants. Synergistic interactions between *apum23* and the adaxial mutants are shown by the partial or complete phloem-surrounding-xylem structure in the double mutants. ph, phloem; x, xylem. Bar, 20 μm (this figure is available in colour at *JXB* online).

**Fig. 5.**
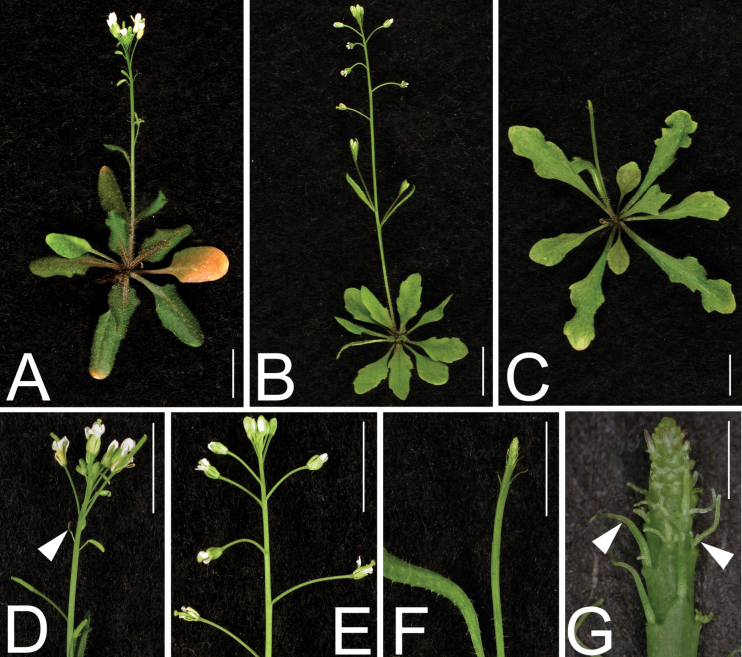
*apum23-3* enhances the inflorescence phenotype of *rev-1*. (A–C) 30-d-old *rev-1* (A), *apum23-3* (B), and *apum23 rev* (C) plants. (D–G) The inflorescences of plants shown in A, B, and C, respectively; G is a higher magnification of F. White arrowhead in D indicates the degenerate flowers in *rev*; white arrowheads in G indicate several of the sterile and filamentous structures in *apum23 rev*. Bars, 1cm (A–C), 1mm (D–F), and 100 μm (G) (this figure is available in colour at *JXB* online).

In addition to enhancing the *kan1 kan2* phenotype, it was also found that *apum23-3* showed synergistic interactions with mutants affecting adaxial fate, such as *revoluta-1* (*rev-1*), *asymmetric leaves1-1* (*as1-1*), and *asymmetric leaves2-2* (*as2-2*)*. REV* encodes a member of the *HD-ZIP III* gene family of transcription factors that redundantly promote adaxial identity (Emery *et al.*, 2003). Due to the overlapping roles of these *HD-ZIP III* genes, the *rev* single mutant did not show strong defects in the vegetative stage (Fig. 3A). However, the *apum23 rev* double mutant had pin-shaped outgrowths in the centre of the rosette that arose after the emergence of several *apum23*-like, but more elongated leaves (Fig. 3B, C). Petiole sections of the expanded leaves of *apum23 rev* double mutants displayed abaxialization of vascular tissues with phloem partially surrounding xylem (Fig. 4D), although the vascular pattern of *rev-1* was similar to that of the wild type (Fig. 4A). Furthermore, *apum23-3* also enhanced the inflorescence defects of *rev*. The *rev* mutant inflorescences contained filamentous organs due to the failure of normal floral organ formation (Talbert *et al.*, 1995; Otsuga *et al.*, 2001; Fig 5A, D). The *apum23-3* mutant strongly enhanced this defect, such that the double mutant displayed a completely sterile inflorescence with filamentous organs (Fig. 5C, F, G).

As well as the HD-ZIPIII genes, *AS1* and *AS2* are also key regulators of adaxial fate. *AS1* encodes a Myb domain transcription factor closely related to the *PHANTASTICA* (*PHAN*) gene product that plays a role in adaxial determination in *Antirrhinum* (Waites and Hudson, 1995; Waites *et al.*, 1998). *AS2* interacts with *AS1* (Xu *et al.*, 2006) and participates in establishing leaf polarity by antagonizing *KAN* genes (Wu *et al.*, 2008). *as1* and *as2* both produce asymmetric, rumpled leaves with ectopic leaflet-like structures on petioles (Tsukaya and Uchimiya, 1997; Byrne *et al.*, 2000; Ori *et al.*, 2000; Semiarti *et al.*, 2001; Sun *et al.*, 2002; Fig. 3 D, G). *apum23-3* displayed similar interactions with *as1* and *as2*. In the *apum23 as1* and *apum23 as2* double mutants, only the first four or five leaves were expanded and the subsequent leaves were replaced by pin-shaped or branched structures (Fig. 3F, I). The expanded leaves were also distinct from those of single mutants in that they occasionally produced a ‘trumpet’ shape (Fig. 3E, H). The vasculature of the petioles was also analysed in these mutants. The *as1* and *as2* mutants often have a slightly abaxialized vein pattern (Ha *et al.*, 2007; Fig. 4B, C). However, in double mutants with *apum23*, the abaxialization was strikingly enhanced. The midveins of these plants were radialized with phloem surrounding xylem (Fig. 4E, F), which is typical of vascular structures completely lacking adaxial identity.

### *apum23-3* displays pleotropic defects in development

**Fig. 6.**
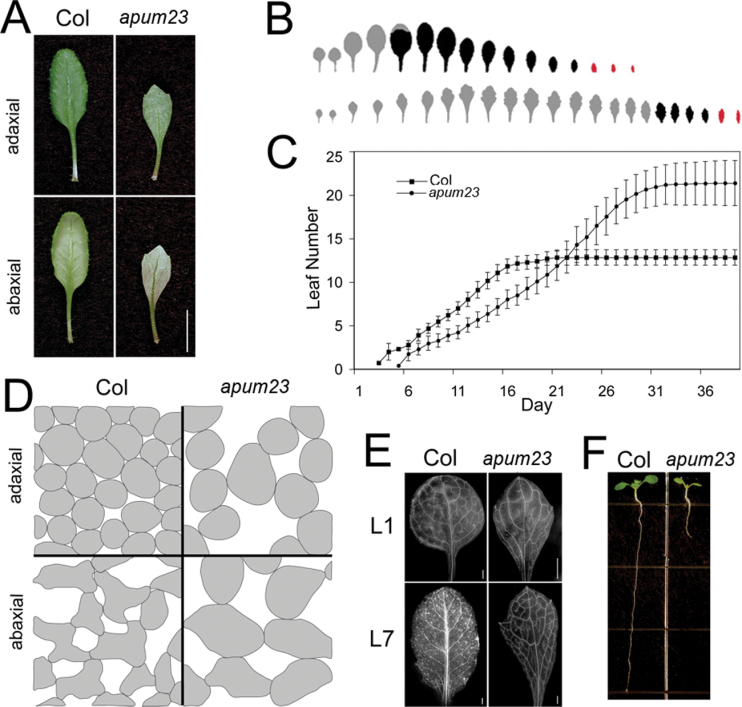
APUM23 plays pleotropic roles in plant development. (A) Adaxial and abaxial surfaces of leaf 5 of 21-d-old Col and *apum23-3* plants. (B) Outline of leaves from Col (upper) and *apum23-3* (lower), indicating the delayed transition from juvenile to adult stage: light grey indicates leaves lacking abaxial trichomes; black indicates leaves with abaxial trichomes; red indicates bracts. (C) The rate of leaf initiation in Col and *apum23-3*: the first visible leaf appears 2 d later in *apum23-3* but leaf production persists longer, resulting in more leaves (*n* = 20). (D) Camera-lucida drawings of the adaxial and abaxial mesophyll layers of leaf 5 in Col and *apum23-3*. (E) Chloral hydrate-cleared leaves of Col and *apum23-3* show reduced complexity of vascular strands in *apum23-3*. (F) Root growth of Col and *apum23-3* on 1/2 MS media. Bar, 1cm (A) and 1mm (E) (this figure is available in colour at *JXB* online).

These strong genetic interactions with key leaf polarity genes imply that APUM23 may play an important role in leaf development. To explore this role, the *apum23-3* single-mutant phenotypes were further characterized. The *apum23-3* homozygous mutant displayed a variety of developmental defects including delayed leaf formation (2 d later than the wild type, Fig. 6C), delayed phase transition from juvenile to adult as measured by the appearance of the first abaxial trichomes (on leaf 17 in *apum23-3* versus leaf 5 in Col; Fig. 6B), and reduced leaf venation complexity (Fig. 6E). The morphology of the subepidermal mesophyll in *apum23-3* was also examined as compared to the wild type (Fig. 6D). In wild-type leaves, adaxial mesophyll cells were round and densely packed, whereas abaxial mesophyll consisted of irregular cells with large air spaces. In *apum23-3*, both adaxial and abaxial mesophyll cells were fewer but slightly bigger than the wild type, and the abaxial cells were more regular in shape. Thus, *apum23* affected the differentiation of both adaxial and abaxial cells in the leaf blade. In addition, *apum23-3* also had shorter roots (0.9±0.1cm) as compared to Col (4.1±0.1cm, Fig. 6F).

### *apum23-3* affects the expression of leaf polarity genes

**Fig. 7.**
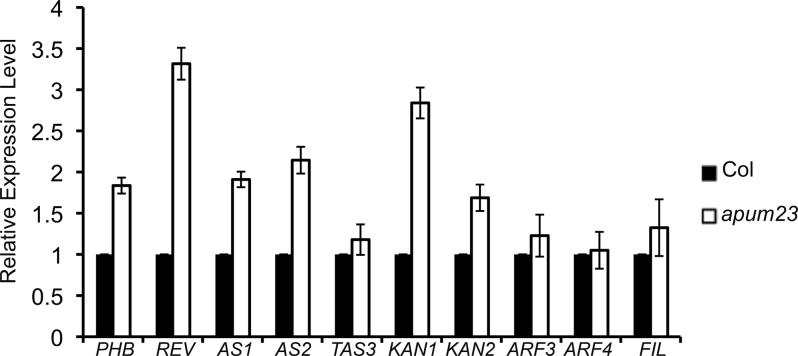
Expression of leaf polarity genes in *apum23-3.* Results of qRT-PCR show the expression of major leaf polarity genes (*PHB*, *REV*, *AS1*, *AS2*, *TAS3*, *KAN1*, *KAN2*, *ARF3*, *ARF4*, and *FIL*), in young leaves of Col (black) and *apum23-3* (white). The relative expression level of genes was normalized by the expression of *GAPC2*. Error bars represent the standard error of the mean.

*APUM23* has been found to be expressed ubiquitously in a variety of organs in *Arabidopsis* (Abbasi *et al.*, 2010), which implies that it is unlikely to have tissue specific functions in leaf polarity. In order to further examine the effect of APUM23 on leaf polarity genes, the expression of *KAN1* and *AS2* was assayed in the wild type and *apum23-3* mutants using promoter:GUS reporter lines. In wild-type seedlings, p*KAN1:GUS* was expressed in the meristem and the abaxial side of young leaf primordia (Supplementary Fig. S2A available at *JXB* online), while p*AS2:GUS* showed a complementary pattern with GUS present only in the adaxial side (Supplementary Fig. S2C available at *JXB* online). The spatial expression patterns of both GUS reporters were not obviously different in the *apum23-3* background (Supplementary Fig. S2B, D available at *JXB* online), indicating that the spatial expression domains of *KAN1* and *AS2* are not affected by *apum23-3.* qRT-PCR was then utilized to measure the mRNA levels of several key leaf polarity genes in the young leaf primordia in the wild type and *apum23-3*. *PHB*, *REV*, *AS1*, *AS2*, *KAN1*, and *KAN2* were all expressed at higher level in *apum23-3* than in the wild type; however, the expression of *TAS3* (ta-siRNA), *ARF3*, *ARF4*, and *FILAMENTOUS FLOWER* (*FIL*, a major member in the *YABBY* gene family) were not significantly altered in *apum23-3* as compared to those in the wild type (Fig. 7). These results indicated that loss of *APUM23* can affect the transcript levels of both adaxial and abaxial regulatory genes, which may be associated with the synergistic phenotypes observed in these double and triple mutant analyses.

### *apum23-3* displays defective cell division patterns in both leaves and roots

**Fig. 8.**
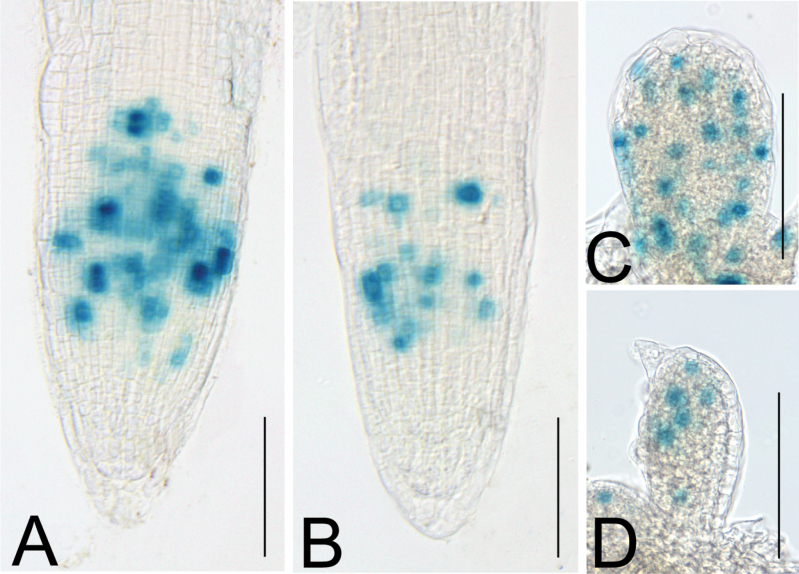
Cell division patterns in Col and *apum23-3*. *CYCB1:db:GUS* expression in roots of Col (A) and *apum23-3*(B) and young leaves of Col (C) and *apum23-3*(D). Bars, 100 μm (this figure is available in colour at *JXB* online).

The *apum23-3* mutant had fewer cells in the mesophyll and shorter roots, suggesting a reduction in cell proliferation. To test this idea, the pattern of cell division was examined in both the wild type and *apum23-3* using the *CYCB1:db:GUS* reporter gene (Harrar *et al.*, 2003). GUS staining showed that the number of division-competent cells was reduced in both leaves and roots of *apum23-3* as compared to the wild type (Fig. 8). The putative function of APUM23 in promoting cell division is consistent with its strong expression in developmentally active tissues (Abbasi *et al.*, 2010) and is likely correlated with the growth defects seen in *apum23* mutants (Fig. 6).

### APUM23 functions in pre-rRNA processing

**Fig. 9.**
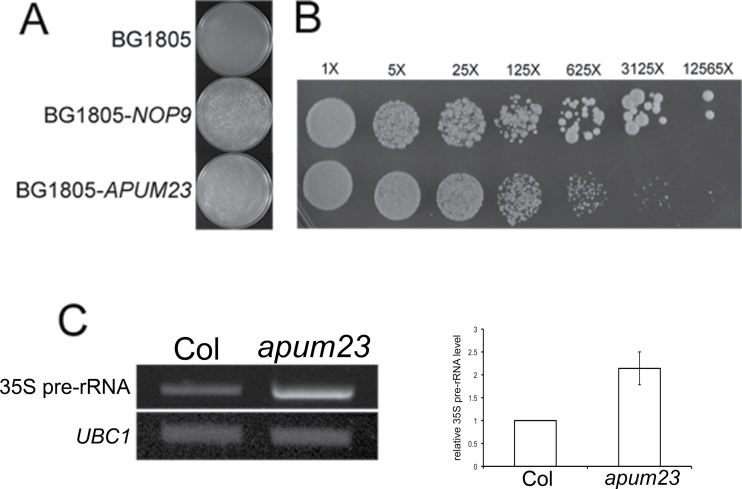
APUM23 functions in 18S rRNA biosynthesis. (A) Colonies of the haploid *nop9* mutant transformed with empty vector BG1805, *NOP9*, and *APUM23*, as indicated. (B) Comparisons of a dilution series of haploid *nop9* mutant cells transformed with *NOP9* (upper) and *APUM23* (lower), plated on Magic Medium. Cells with *APUM23* form smaller colonies than those with *NOP9*. Fold-dilution is indicated above each column. (C) Enrichment of unprocessed 35S pre-RNA in *apum23-3* is revealed by semiquantitative reverse-transcription PCR. Error bar represents the standard error of the mean. *UBC1* serves as the loading control.

APUM23 belongs to the *Pumilio/PUF* gene family that is evolutionarily conserved across kingdoms (Spassov and Jurecic, 2003; Abbasi *et al.*, 2010). The APUM23 protein has been found to play a specific role in the cleavage of 35S pre-rRNA, a critical step for 18S rRNA biosynthesis (Abbasi *et al.*, 2010). This function is similar to that of *NOP9*, a *PUF* gene in *Saccharomyces cerevisiae* (Thomson *et al.*, 2007; Abbasi *et al.*, 2010). In order to further confirm the functional homology of APUM23 and NOP9, a complementation experiment was performed in yeast cells. Because the null *nop9* mutant is lethal, a heterozygous diploid strain, *nop9/+*, was transformed with *NOP9* or *APUM23* full-length cDNA, and Magic Marker technology (Pan *et al.*, 2004) was utilized to select the *nop9* haploid cells carrying *NOP9* or *APUM23* after sporulation. Growth tests showed that *APUM23* could partially rescue *nop9* defects (Fig. 9A, B), which suggested some level of functional similarity between these two proteins. Because the cleavage of 35S pre-rRNA is conserved in eukaryotes (Venema and Tollervey, 1999) and the cleavage site in *Arabidopsis* has been identified in previous studies (Saez-Vasquez *et al.*, 2004; Shi *et al.*, 2005), the accumulation of the unprocessed 35S pre-RNA was examined in the wild type and *apum23-3* using established methods (Petricka and Nelson, 2007). As expected, the amount of unprocessed 35S rRNA was 2.1-fold higher in *apum23-3* as compared to the wild type (Fig. 9C). These results further support and extend the observation that APUM23 plays a critical role in 35S pre-rRNA processing (Abbasi *et al.*, 2010).

## Discussion

### APUM23 is a unique PUF protein involved in rRNA biosynthesis and functions in regulating cell division in *Arabidopsis*

The *Arabidopsis* genome encodes more than 20 putative PUF proteins (Francischini and Quaggio, 2009; Abbasi *et al.*, 2010; Tam *et al.*, 2010). Among all these PUF family members, APUM23 is the only one that possesses PUF repeats outside the conserved C-terminal PUM-HD region (Tam *et al.*, 2010). Consistent with this unusual structural feature, the major molecular function of APUM23 in regulating 35S pre-rRNA processing (Abbasi *et al.*, 2010; this study) is also distinct from those of other *PUF* genes in *Arabidopsis* (Francischini and Quaggio, 2009).

Other *Arabidopsis* mutants disrupting rRNA synthesis also have morphological and developmental defects similar to the phenotypes of *apum23* alleles (Abbasi *et al.*, 2010; this study), such as narrow and pointed leaves, defective vein patterning, and reduced root growth and leaf initiation (Shi *et al.*, 2005; Kojima *et al.*, 2007; Petricka and Nelson, 2007). These growth defects have been proposed to be associated with impaired cell division in plants (Petricka and Nelson, 2007). In the current study, the cell division patterns in *apum23* mutants were characterized using *CYCB1:db:GUS* and the results showed that cell division activity is indeed reduced in both roots and leaves of *apum23-3* (Fig. 8). This reduction in cell division is likely associated with the lower number of mesophyll cells in the leaf blade, which results in the pale green colour of the mutant leaves (Fig. 6). These mesophyll cells are also slightly bigger than those of the wild type probably due to ‘compensation’ mechanisms that coordinate cell number and cell size in an organ (Ferjani *et al.*, 2007; Fig. 6). Reduced cell division is also possibly related to the narrow and flat leaves in *apum23-3*, because growth repression results in leaves with reduced curvature and blade expansion (White, 2006). In addition, the short root phenotype is also likely attributable to the reduced activity of cell division zone (Fig. 6). Defective cell division seems to be tightly correlated with the major growth abnormalities observed in the *apum23-3* mutant, so clarifying the genetic mechanisms that account for the reduction in cell division may help lead to a better understanding of the regulation of organ growth by APUM23.

### Role of APUM23 in the regulation of leaf polarity

The *apum23-3* mutant on its own displayed a mild leaf polarity defect. There was a modestly decreased distinction between the adaxial and abaxial mesophyll (Fig. 6), but vascular structures did not differ noticeably from the wild type (Fig. 1). However, mRNAs of a number of polarity genes (*PHB*, *REV*, *AS1*, *AS2*, *KAN1*, *KAN2*) accumulated to a higher level in the *apum23* mutants than in the wild type during early stages of leaf development (Fig. 7), which suggests that APUM23 does influence the expression of these critical leaf polarity regulators. Normal leaf polarity is highly dependent on the balance between antagonistic adaxial- and abaxial-specifying genes. In the *apum23* mutant, this balance may not be strongly altered because both types of key regulators are simultaneously upregulated, so the defects in leaf polarity are relatively mild. However, if one or more of these key genes are also mutated, the balance will be altered and proper polarity formation may be disrupted to an even greater degree, which may explain the strong polarity defects observed in double and triple mutants with *apum23*.

The major molecular function of APUM23 is to regulate rRNA biosynthesis, which acts primarily on the translational machinery (Abbasi *et al.*, 2010; this study), but how APUM23 controls the transcription of leaf polarity genes is still not understood. One plausible mechanism is that APUM23 may act indirectly on leaf polarity genes through translational regulation of genes that, in turn, control the transcription of leaf polarity genes. Alternatively, it may be possible that APUM23 also regulates the biogenesis of other RNA species, such as small RNAs, required for leaf polarity. It has been reported that genes involved in rRNA processing are also required for miRNA biogenesis (Fukuda *et al.*, 2007). In leaf polarity, miRNA play important roles to posttranscriptionally regulate critical genes that APUM23 interacts with, such as *HD-ZIP III* genes (Bao *et al.*, 2004; Juarez *et al.*, 2004; Kidner and Martienssen, 2004; Mallory *et al.*, 2004). Interestingly, *HD-ZIP III* genes also have similar synergistic interactions with genes encoding subunits of ribosome proteins that interact with rRNA to constitute the ribosome (Pinon *et al.*, 2008; Yao *et al.*, 2008). It has been hypothesized that these ribosomal genes may interact with the small RNA silencing complex RISC to mediate miRNA or siRNA functions, which in turn affect the function of leaf polarity genes (Pinon *et al.*, 2008). Given that the phenotypes of the single mutants of these ribosomal genes and their double mutants with leaf polarity genes both resemble those of *apum23* (Pinon *et al.*, 2008; Yao *et al.*, 2008), it is possible that these two essential components of the ribosome may act in related posttranscriptional pathways to control the expression of leaf polarity genes. Future studies to identify the proteins that interact with APUM23 will hopefully provide more insights into this possibility and help to unravel further the function of this unique type of PUF protein in *Arabidopsis*.

## Supplementary material

Supplementary data are available at *JXB* online.

Supplementary Fig. 1. Phenotype of the confirmed T-DNA insertion line of *APUM23* SAIL_757_B08 (*apum23-1*).

Supplementary Fig. 2. Spatial expression patterns of *KAN1* and *AS2* in Col and *apum23-3*.

Supplementary Table S1. Primers used in qRT-PCR.

Supplementary Data
