# Supplementary material for: APUM23, a PUF family protein, functions in leaf development and organ polarity in Arabidopsis
Source: J Exp Bot. 2014 Jan 21;65(4):1181–91. doi: 10.1093/jxb/ert478 (PMC3935572; doi:10.1093/jxb/ert478)
Supplement: Supplementary Data [file supp_65_4_1181__index.html]

APUM23, a PUF family protein, functions in leaf development and organ polarity in Arabidopsis — APUM23, a PUF family protein, functions in leaf development and organ polarity in Arabidopsis — Supplementary Data 

# APUM23, a PUF family protein, functions in leaf development and organ polarity in *Arabidopsis*

## Supplementary Data

Data files

**Files in this Data Supplement:**

- Supplementary Data - Supplementary Data
