# Supplementary material for: APUM23, a PUF family protein, functions in leaf development and organ polarity in Arabidopsis
Source: J Exp Bot. 2014 Jan 21;65(4):1181–91. doi: 10.1093/jxb/ert478 (PMC3935572; doi:10.1093/jxb/ert478)
Supplement: Supplementary Data [file supp_ert478_jexbot114728_file001.pdf]

Supplementary Figure 1. Phenotype of the confirmed T-DNA insertion line of *APUM23*, SAIL_757_B08 (*apum23-1*). Note that SAIL_757_B08 (right) has pale green and pointed leaves as compared to Col (left), which resembles *apum23-3*.

Supplementary Figure 2. Spatial expression patterns of *KAN1* and *AS2* in Col and *apum23-3*. Promoter:GUS reporters show the spatial expression of *KAN1* in Col (**A**) and *apum23-3* (**B**), and that of *AS2* in Col (**C**) and *apum23-3*(**D**). Bar=50µm

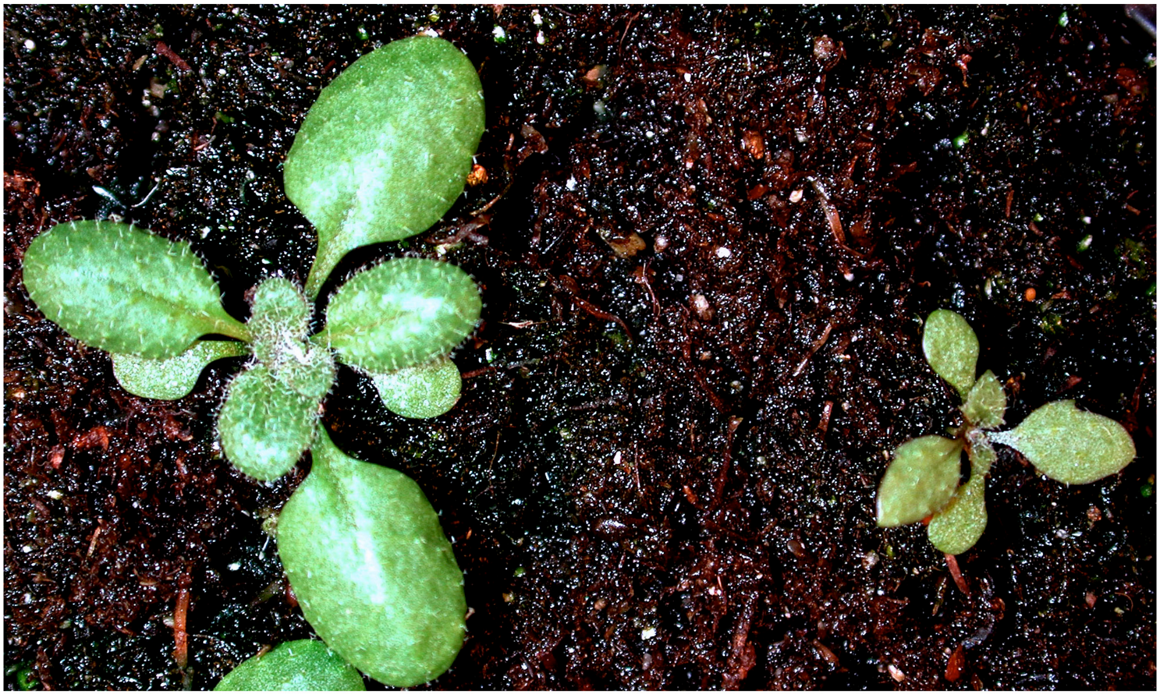

Col SAIL_757_B08

Suppl. Figure 1

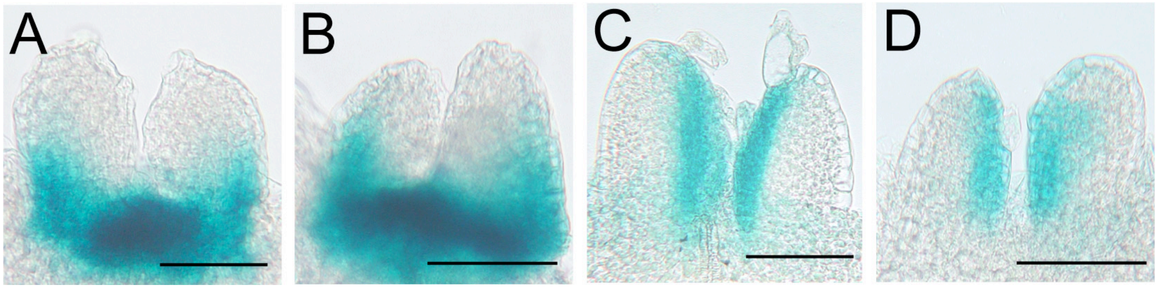

Suppl. Figure 2

Supplementary Table

Gene	Forward primer	Reverse primer
GAPC2	ctgttgggaaagtgttgccatccctcaat	catgtgaacgataaggtcaacgacacga
PHB	ctggtgaagccgacccaaatggctgt	tggagccaggacgaggagcaatgg
REV	tgcaatggcgatctcaccgtctgg	accgagtcgtcgcttccaagtgaat
AS1	gctgcgcctcaaccgccaatcc	tgcccaagctcggtgcccttcc
AS2	gccgacatgcgcctccgtga	tgatgagtggcggcgaggataccg
TAS3	gaaagagagagaagagctcccatgg	ccgactaaaagctcagataggataacac
KAN1	agcacatgtgaagagccatttgcag	tgtgccctttgatccgtcgatgattg
KAN2	gtggccatgaaagagcaacacc	ctgctttgtcggttgtcttcac
ARF3	ggtggagccggtgccaggaga	acccgtgctgctgccgatga
ARF4	ggcatgatttctgcaatgtggtgtgg	ggagaagaatcaggctggctcacagaa
FIL	gcaccacccgttaaccgccctcca	ttgtctggcacgagcccgaagtg
